# Lowly polymorphic pathogen proteins may not be under evolutionary constraint

**DOI:** 10.64898/2026.10.14.759147

**Published:** 2026-10-20

**Authors:** Ana Pinharanda, Sarah A Perkins, Angela M Early, Daniel E Neafsey

## Abstract

A central challenge in infectious disease biology is anticipating whether pathogen proteins targeted by vaccines, drugs, or diagnostics can evolve in ways that reduce intervention efficacy. Evolutionary constraint is therefore relevant to selecting targets likely to remain effective after deployment. Yet low diversity in contemporary pathogen populations is often interpreted as evidence of evolutionary constraint, an inference that can be misleading. In this Personal View, we use *Plasmodium falciparum* malaria to illustrate how contemporary population diversity and longer-term cross-species divergence can inform assessments of evolutionary constraint. A severe population bottleneck reduced diversity across the *P. falciparum* genome, meaning that low diversity in a particular protein-coding gene does not necessarily indicate unusual constraint. We place gene-level diversity in its genome-wide context, compare it with divergence across related ape-infecting malaria parasites, and examine how both relate to experimental essentiality. We then examine diversity and divergence along vaccine, drug, and diagnostic targets, considering them in relation to known resistance-associated mutations and other routes of evolutionary escape. We introduce PEEP (Protein Evolution Explorer for Plasmodium), a public interactive resource for exploring diversity and divergence across *P. falciparum* protein-coding genes and along individual proteins. Although our analyses focus on *P. falciparum* malaria, the implications extend across pathogens: low diversity alone is insufficient evidence of evolutionary constraint, and assessments of intervention durability should consider constraint alongside potential routes of evolutionary escape.

## Full Text Availability

The license terms selected by the author(s) for this preprint version do not permit archiving in PMC. The full text is available from the preprint server.

